# Reading activities compensate for low education-related cognitive deficits

**DOI:** 10.1186/s13195-022-01098-1

**Published:** 2022-10-14

**Authors:** Yue Wang, Shinan Wang, Wanlin Zhu, Na Liang, Chen Zhang, Yuankun Pei, Qing Wang, Shiping Li, Jiong Shi

**Affiliations:** 1 Department of Neurology, Beijing Tiantan Hospital, Capital Medical University, Beijing, China; 2 National Clinical Research Center for Neurological Diseases, Beijing Tiantan Hospital, Capital Medical University, Beijing, China; 3 Advanced Innovation Center for Human Brain Protection, Capital Medical University, Beijing, China; 4 Department of Neurology, Hebei Yanda Hospital, Sanhe, Hebei Province China

**Keywords:** Reading activities, Education, Cognition

## Abstract

**Background:**

The incidence of cognitive impairment is increasing with an aging population. Developing effective strategies is essential to prevent dementia. Higher education level is associated with better baseline cognitive performance, and reading activities can slow down cognitive decline. However, it is unclear whether education and reading activities are synergistic or independent contributors to cognitive performance.

**Methods:**

This was a sub-study of an ongoing prospective community cohort of China National Clinical Research Center Alzheimer’s Disease and Neurodegenerative Disorder Research (CANDOR). Demographic and clinical information, educational levels, and reading activities were collected. All participants finished neuropsychological testing batteries and brain MRIs. We analyzed cognitive performance and brain structures with education and reading activities.

**Results:**

Four hundred fifty-nine subjectively cognitively normal participants were enrolled in the study. One hundred sixty-nine (36.82%) of them had regular reading activities. Participants in the reading group had better performance in all cognitive tests compared with those in the non-reading group, but no difference in brain MRI variables. Participants with higher education levels (more than 13 years) had better cognitive performance and higher hippocampal volumes. In low education groups (less than 12 years), more reading activities were associated with better cognitive test scores.

**Conclusions:**

Both education and reading activities are important and synergistic for baseline cognitive function. Higher education level is associated with larger hippocampal volumes. Education may stimulate the growth and development of the hippocampus. Reading activities help to maintain and improve cognitive function in people with low levels of education.

**Trial registration:**

NCT04320368.

**Supplementary Information:**

The online version contains supplementary material available at 10.1186/s13195-022-01098-1.

## Introduction

Aging is the most important risk factor for dementia. With an aging population, dementia has cast an enormous social and economic burden around the world [1, 2]. Developing effective strategies is essential to prevent dementia [3]. It has been reported there are modifiable risk factors for dementia and modifying 12 of them may prevent or delay up to 40% of dementia [4]. The Finnish Geriatric Intervention Study to Prevent Cognitive Impairment and Disability (FINGER), a multicenter randomized controlled trial, reported beneficial effects on cognition through multimodal intervention including cognitive training [5]. Reading activities and other mental stimulation help to slow down cognitive decline [6, 7].

High education level is associated with better cognitive performance and lower likelihood to have Alzheimer’s disease (AD) [8, 9]. High education level may delay cognitive decline in individuals with subjective cognitive decline [10, 11]. However, although education is associated with baseline cognitive performance, it doesn’t affect the rate of cognitive decline [12], nor does it affect the neuropathological changes related to dementia, such as amyloid plaques and tangles [13].

Previous research compared the influence of reading activities and education on cognition and found that reading activities were associated with a lower risk of dementia even in late life, independent of education and other related factors [6, 7], while another study demonstrated that reading activities have a stronger relationship than education with executive function tests [14].

It is inconclusive whether reading activities and education are synergistic or independent contributors to cognitive performance. In this prospective community-based cohort study, we try to answer the following questions. First, what are the relationships of education and reading activities with cognitive performance on domain-specific tests? Second, are education and/or reading activities associated with brain structure? Third, can reading activities compensate for lower levels of education?

## Methods

### Study design and participants

This study was a sub-study of an ongoing prospective community-based cohort study of the China National Clinical Research Center Alzheimer's Disease and Neurodegenerative Disorder Research (CANDOR). CANDOR was started in July 2019 and planned to enroll one thousand and five hundred participants, including individuals with normal cognition (NC), mild cognitive impairment (MCI), and dementia. Demographic information and past medical history were collected. All participants were required to have a study partner to provide an independent evaluation of daily and social functions. They underwent detailed assessments for cognition and functional abilities, a comprehensive neuropsychological battery (described below in “Neuropsychological assessment”) including the Mini-Mental State Examination (MMSE), Clinical Dementia Rating (CDR), and brain MRIs. All enrolled participants for this study (1) were subjectively cognitively normal; (2) aged 40–100 years old; (3) had at least 3 years of education; (4) had no condition known to affect cognitive function, such as Alzheimer’s Disease, vascular dementia, Lewy body dementia, frontotemportal dementia, Parkinson’s disease, epilepsy, stroke, hydrocephalus, multiple sclerosis, traumatic brain injuries, genetic disorders affecting cognition, alcoholism, uncontrolled depression, or other psychiatric disorders; (5) had no uncontrolled neoplasia, or severe pulmonary, cardiovascular, metabolic, infectious, inflammatory, or endocrine diseases. We excluded individuals with less than 3 years of education because people started to learn how to read and write in the third year of elementary school in China. Therefore, people who have less than three years of education will have difficulties in reading.

To assess the relationship between education and leisure reading activities, we defined regular reading activities as reading at least one book per month on average for at least one year. We divided the participants as follows. First, participants were divided into 2 groups based on their reading activities. Reading activities were detailed, including (1) reading materials, such as paper books, e-books, and audio-books; (2) reading content, such as literature books, and non-literature books; (3) the total number of books, which was calculated as the average number of books read per month ×12 months × years of reading. In participants with reading habits, we divided them further into several groups based on reading years, reading content and reading materials. Second, participants were divided based on their education. Previous studies analyzed education by ≤9, 10–12, and ≥13 years [15, 16]. In our study, the average education years of all participants were 12.12 years. Therefore, we used a 12-year cut-off to divide participants into two groups: low education (≤12 years, high school education or below, under the average education level) and high education (≥13 years, college education or above, over the average education level). Third, participants were divided into four groups based on education years and reading activities: low education (educational years ≤12) with and without reading activities (groups 1 and 2), and high education (educational years ≥13) with and without reading activities (groups 3 and 4).

### Standard protocol approvals, registrations, and patient consents

This protocol was approved by the Institutional Review Board of Beijing Tiantan Hospital (approval number: KY 2019-004-007) and was in accordance with relevant guidelines and regulations. Written informed consent was obtained from each participant.

### Neuropsychological assessment

Thirteen neuropsychological tests were completed at the visit, including (1) tests for overall cognitive performance: Mini-Mental State Examination (MMSE), Montreal Cognitive Assessment (MoCA), and Clinical Dementia Rating (CDR) with global scores; (2) Tests for specific cognitive domain: Rey Auditory Verbal Learning Test (RAVLT) [17], Rey-Osterrieth Complex Figure Test (ROCF) [18], Stroop Color-Word Test-Victoria version [19], Trail Making Test-A (TMT- A) and Trail Making Test B (TMT -B) [20], clock drawing test (CDT), Boston Naming Test (BNT), Digit Span Test (DST), and Symbol Digit Modalities Test (SDMT); and (3) Neuropsychiatry Inventory (NPI). These tests were administered by experienced neuropsychologists who were blinded to group assignment.

### MRI assessment

All participants completed the brain MRI to exclude other demonstrable neurological diseases. Quantitative measures of signal-to-noise ratio, uniformity, and geometric distortion were conducted in each research center. 3.0 T-MRI was used with the scanning thickness not exceeding 1.5mm. The three-dimensional T1 weighted images were corrected for intensity non-uniformity with the N4 algorithm. Brain surface was reconstructed using FreeSurfer (version 7.2.0, http://surfer.nmr.mgh.harvard.edu/) recon-all pipeline. The cortical thickness and volume of the total brain, nuclei, gray matter white matter, and white matter lesion were all obtained with this pipeline. Regional cortical thickness was obtained and statistical analysis was performed.

### Statistical analysis

The analysis was conducted with SPSS 24.0. Continuous variables were characterized as mean plus and/or minus standard deviations (SD). *T*-test or nonparametric test was used by the characteristic of the distribution. Categorical variables were analyzed by Pearson’s *χ*^2^ tests. We performed logistic regression analysis to evaluate the association between reading and CDR (CDR=0 or >0), linear regression analysis for the association of reading and neuropsychological assessment, and linear regression analysis for education and brain structure. The regression analyses were independent of age and sex in Table 2 model 1 and Table 5. The regression analyses were independent of age, sex, and education in Table 2 model 2. We also performed the collinearity analysis in every linear regression analysis, and all the results showed no collinearity between every included independent variable.

## Results

**Fig. 1:**
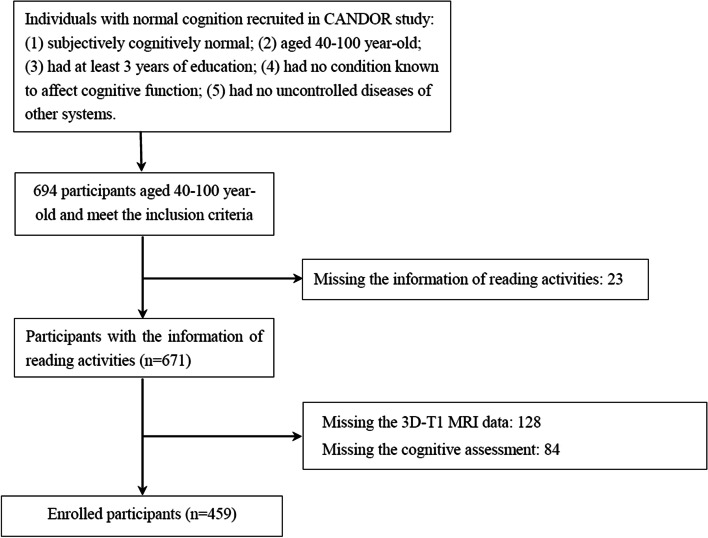
Study flowchart. Shown is the flowchart of the study enrollment

From July 31, 2019, to August 1, 2021, 694 individuals were screened from communities in Beijing, Shijiazhuang, and Langfang, all in northern China. They completed standard baseline assessments. 459 were enrolled in the study who had both valid brain MRI examination and cognitive evaluation (Fig. 1). Among them, 169 (36.82%) had regular reading activities. There was no significant difference in age, sex, medical history, and mood assessment (NPI) between the two groups (Table 1). The reading group had more years of education and better cognitive performance than the non-reading group, including CDR, MMSE, MoCA, DST, RAVLT, ROCF, Stroop D and W time, TMT-A and B, BNT, SDMT, and CDT. However, there was no difference in cortical thickness and hippocampal volume in either hemisphere between the two groups.

**Table 1:** Demographic, clinical information, cognitive test scores, and MRI variables in reading and non-reading groups

	Reading *n*=169	Non-reading *n*=290	All patients *n*=459	*P* value
Average age	60.33±9.04	60.03±8.5	60.14±8.69	0.727
Sex female, (*n*, %)	89, 52.7%	171, 59.0%	260, 56.6%	0.189
Years of education	13.88±3.3	11.1±3.52	12.12±3.69	<0.001
Hypertension (*n*, %)	57, 33.7%	96, 33.1%	153, 33.3%	0.918
Diabetes (*n*, %)	19, 11.2%	34, 11.7%	53, 11.5%	0.882
Stroke or TIA (*n*, %)	11, 6.5%	21, 7.2%	32, 7.0%	0.851
Coronary heart disease (n, %)	14, 8.3%	18, 6.2%	32, 7.0%	0.449
Global CDR score	0.11±0.21	0.22±0.41	0.18±0.36	0.001
MMSE	26.15±2.85	24.04±4.82	24.82±4.32	<0.001
MoCA	23.38±3.82	20.13±5.64	21.33±5.29	<0.001
DST total	12.3±2.34	11.09±2.93	11.54±2.79	<0.001
RAVLT total learning	39.98±10.06	36.26±12.68	37.63±11.91	0.001
RAVLT long-delayed recall	8.01±3.44	6.85±3.85	7.28±3.74	0.001
ROCF copy	32.34±6.91	28.48±10	30.36±8.83	0.001
ROCF long-delayed recall	16.5±7.82	11.03±9.01	13.69±8.87	<0.001
Stroop D time	16.86±6.13	21.8±16.31	19.97±13.67	<0.001
Stroop W time	22.32±8.05	26.77±14.46	25.12±12.65	<0.001
TMT-A time	44.18±21.57	58.17±35.98	52.98±32.11	<0.001
TMT-B time	101.55±71.89	125.32±87.41	116.51±82.72	0.002
BNT	25.02±3.4	21.99±4.37	23.11±4.29	<0.001
SDMT	39.57±13.39	32.71±14.89	35.25±14.72	<0.001
CDT	8.79±1.79	8.09±2.42	8.35±2.23	<0.001
NPI	1.03±3.17	1.83±6.01	1.54±5.15	0.064
Brain structure				
Left hippocampal volume, mm^2^	3496.15±427.05	3436.04±451.65	3458.79±442.97	0.170
Left amygdala volume, mm^2^	1463.18±355.93	1482.53±324.29	1475.21±336.34	0.561
Left thalamus volume, mm^2^	6879.88±904.81	6876.82±939.2	6877.98±925.29	0.973
Left caudate volume, mm^2^	3316.52±565.36	3297.41±581.55	3304.64±574.9	0.737
Left putamen volume, mm^2^	4632.86±662.24	4613.26±719.29	4620.68±697.55	0.776
Left pallidum volume, mm^2^	1940±269.78	1916.5±238.56	1925.39±250.78	0.343
Left cortex volume, mm^2^	213,629.66±24,614.53	215,780.91±24,480.75	214,966.79±24,525.43	0.375
Left cerebral white matter volume, mm^2^	221,073.68±32,180.9	222,387.88±29,129.85	221,890.53±30,290.75	0.661
Left mean cortical thickness, mm	2.37±0.11	2.38±0.12	2.37±0.11	0.516
Right hippocampal volume, mm^2^	3605.87±487.08	3571.79±460.36	3584.69±470.39	0.464
Right amygdala volume, mm^2^	1643.52±356.73	1664.16±329.83	1656.35±340	0.539
Right thalamus volume, mm^2^	6643.18±897.79	6669.33±823.35	6659.44±851.36	0.756
Right caudate volume, mm^2^	3366.34±617.01	3358.4±559.61	3361.41±581.31	0.869
Right putamen volume, mm^2^	4714.73±647.17	4743.82±707.15	4732.81±684.46	0.667
Right pallidum volume, mm^2^	1937.36±281.85	1920.2±253.02	1926.7±264.11	0.511
Right cortex volume, mm^2^	212,619.37±25,297.4	215,773.47±24,519.75	214,579.83±24,835.2	0.199
Right cerebral white matter volume, mm^2^	219,923.7±31,876.29	221,611.7±28,804.31	220,972.9±29,978.85	0.569
Right mean cortical thickness, mm	2.36±0.11	2.37±0.12	2.37±0.11	0.151
Cortex volume, mm^2^	426,249.03±49,571.32	431,554.38±48,668.99	429,546.62±49,023.28	0.274
Subcortex gray volume, mm^2^	54,301.14±6096.21	54,392.28±6088.1	54,357.79±6084.32	0.88
Total gray volume, mm^2^	440,997.39±63,865.8	443,999.58±57,770.04	442,863.43±60,094.68	0.613
Cerebral white matter volume, mm^2^	3788.28±5597.58	2941.34±4143.9	3261.86±4758.19	0.093
WM hyperintensities volume, mm^2^	578,959.97±62,023.02	584,269.48±59,903.83	582,260.15±60,698.27	0.376
Brain segmentation volume, mm^2^	1,077,237.37±118,406.82	1,084,041.34±113,069.4	1,081,466.44±115,030.46	0.488
eTIV, mm^2^	1,430,023.25±162,478.34	1,438,793.2±151,423.56	1,435,474.3±155,572.46	0.499
Brain segmentation volume to eTIV, %	75.59±5.81	75.55±5.38	75.56±5.54	0.941

*Abbreviations*: *CDR* Clinical Dementia Rating, *MMSE* Mini-Mental State Examination, *MoCA* Montreal Cognitive Assessment, *DST* Digit Span Test, *RAVLT* Rey Auditory Verbal Learning Test, *ROCF* Rey-Osterrieth Complex Figure Test, *TMT* Trail Making Test, *BNT* Boston Naming Test, *SDMT* Symbol Digit Modalities Test, *CDT* clock drawing test, *NPI* Neuropsychiatry Inventory, *WM* white matter, *eTIV* estimated total intracranial volume

Logistic and linear regression were used to assess confounding factors (Table 2). Reading activities were associated with better cognitive performance, such as MMSE (beta 2.193, 95%CI: 1.463–2.923, *P*<0.001), independent of age and sex in model 1. In model 2, when education was taken into account, reading activities showed similar effects in MoCA and Boston Naming; significant but less effects in MMSE, DST, ROCF delayed recall, Stroop D and W time, TMT-A and SDMT, but no effects in CDR, RAVLT, ROCF copy, TMT-B, and CDT.

**Table 2:** The logistic and linear regression analysis of reading in all cognitive tests

Logistic regression	Model 1, OR, 95% CI	*P*	Model 2, OR, 95% CI	*P*
Global CDR score (=0), 330 (71.9%)	2.012, 1.258–3.218	0.004	1.416, 0.848–2.365	0.210
Linear regression	Model 1, Beta, 95%CI	P	Model 2, Beta, 95%CI	P
MMSE	2.193 [1.463, 2.923]	<0.001	1.044 [0.302, 1.787]	0.006
MOCA	3.342 [2.486, 4.198]	<0.001	1.546 [0.744, 2.348]	<0.001
DST total	1.240 [0.749, 1.731]	<0.001	0.496 [0.003, 0.988]	0.048
RAVLT learn total	4.162 [2.208, 6.121]	<0.001	1.235 [−0.758, 3.227]	0.224
RAVLT long-delayed recall	1.185 [0.544, 1.826]	<0.001	0.241 [−0.426, 0.907]	0.478
ROCF copy	3.800 [1.487, 6.114]	0.001	1.296 [−0.998, 3.590]	0.267
ROCF delayed recall	5.359 [3.138, 7.580]	<0.001	3.103 [0.892, 5.314]	0.006
Stroop D time	−5.162 [−7.712, −2.611]	<0.001	−3.080 [−5.789, −0.371]	0.026
Stroop W time	−4.802 [−7.074, −2.530]	<0.001	−3.304 [−5.694, −0.914]	0.007
TMT-A time	−14.499 [−19.792, −9.206]	<0.001	−8.246 [−13.798, −2.694]	0.004
TMT-B time	−26.290 [−40.525, −12.055]	<0.001	−7.465 [−22.449–7.519]	0.328
BNT	2.961 [2.252, 3.669]	<0.001	1.761 [1.048, 2.474]	<0.001
SDMT	7.385 [5.233, 9.538]	<0.001	2.719 [0.693, 4.746]	0.009
CDT	0.703 [0.284, 1.121]	0.001	0.219 [−0.218, 0.656]	0.325

Model 1 logistic or linear regression included age and sex

Model 2 logistic or linear regression included age, sex, and years of education

*Abbreviations*: *OR* odds ratio for logistic regression, *CI* confidence interval, *CDR* Clinical Dementia Rating, *MMSE* Mini-Mental State Examination, *MoCA* Montreal Cognitive Assessment, *DST* Digit Span Test, *RAVLT* Rey Auditory Verbal Learning Test, *ROCF* Rey-Osterrieth Complex Figure Test, *TMT* Trail Making Test, *BNT* Boston Naming Test, *SDMT* Symbol Digit Modalities Test, *CDT* clock drawing test, *NPI* Neuropsychiatry Inventory

**Fig. 2:**
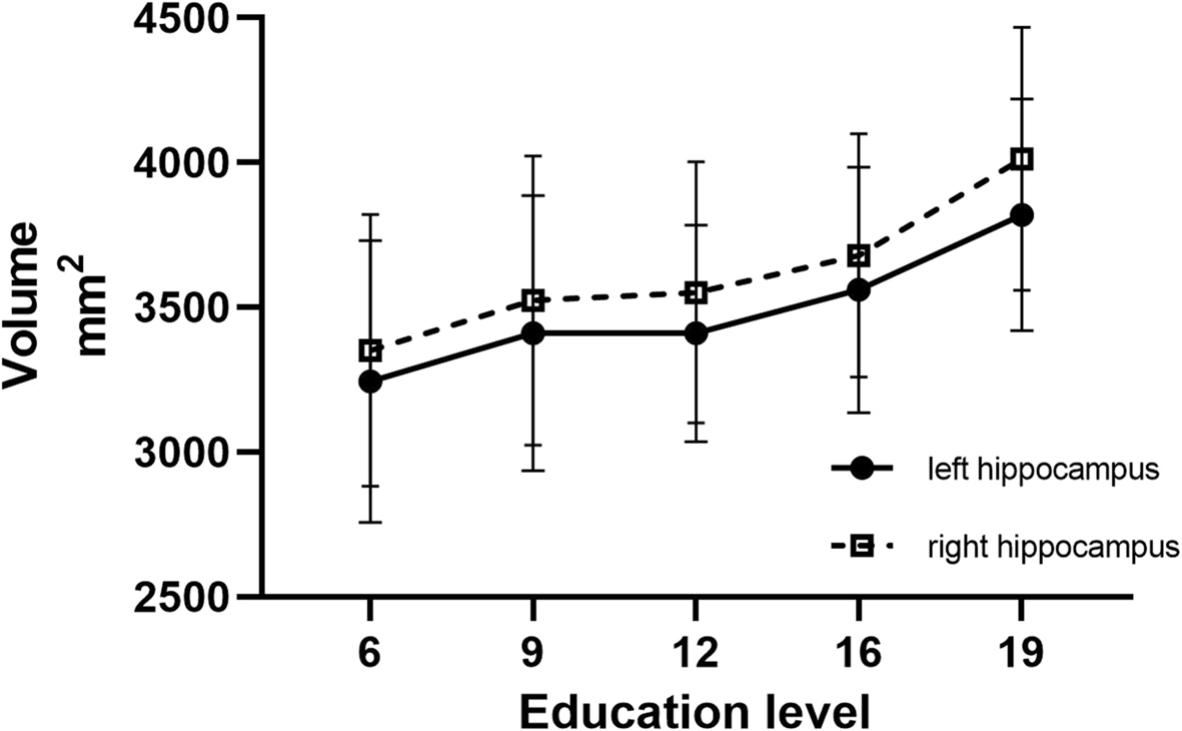
Hippocampal volumes at different education levels. Shown is the right (open box) and left (closed dot) hippocampal volume associated with different education levels

Education had a remarkable effect on cognitive performance (Table 3). Participants with high education scored higher in all cognitive tests than those with low education. They also have higher hippocampal volumes on both sides (Fig. 2).

**Table 3:** Cognitive performance and brain structure at different education levels

	Education ≤12 years *n*=294	Education ≥13 years *n*=165	*P*
Average age	61.66±8.28	57.43±8.78	<0.001
Sex female (*n*, %)	177, 60.2%	83, 50.3%	0.040
Years of education	9.79±2.34	16.29±0.95	<0.001
Global CDR score	0.10±0.24	0.22±0.40	<0.001
MMSE	23.74±4.65	26.73±2.79	<0.001
MoCA	19.47±5.18	24.62±3.61	<0.001
DST total	10.72±2.51	12.98±2.68	<0.001
RAVLT total learning	8.93±3.32	11.07±2.79	<0.001
RAVLT long-delayed recall	6.04±3.7	8.65±3.53	<0.001
ROCF copy	33.18±6.55	28.8±9.53	<0.001
ROCF long-delayed recall	17.23±7.96	11.74±8.76	<0.001
Stroop D time	22.07±16.02	16.25±6.55	<0.001
Stroop W time	27.72±13.85	20.54±8.46	<0.001
TMT-A time	59.73±32.17	41.09±28.42	<0.001
TMT-B time	136.48±91.03	81.31±48.78	<0.001
BNT	21.78±4.47	25.44±2.69	<0.001
SDMT	30.22±13.05	44.06±13.285	<0.001
CDT	8.00±2.39	8.96±1.76	<0.001
NPI	1.75±5.86	1.16±3.55	0.242
Brain structure			
Left hippocampal volume, mm^2^	3386.57±435.93	3584.61±428.01	<0.001
Left amygdala volume, mm^2^	1634.78±220.55	1709.65±215.06	0.145
Left thalamus volume, mm^2^	6883.74±897.91	6867.95±973.97	0.864
Left caudate volume, mm^2^	3313.07±580.16	3289.96±567.13	0.697
Left putamen volume, mm^2^	4629.44±710.25	4605.42±676.78	0.73
Left pallidum volume, mm^2^	1931.57±251.23	1914.63±250.43	0.498
Left cortex volume, mm^2^	216,139.25±23,700.57	212,924.21±25,849.25	0.188
Left cerebral white matter volume, mm^2^	223,576.87±29,777.23	218,952.71±31,041.08	0.125
Left mean cortical thickness, mm	2.37±0.12	2.38±0.10	0.678
Right hippocampal volume, mm^2^	3512.3±476.23	3710.8±433.37	<0.001
Right amygdala volume, mm^2^	4691.13±49,986.08	1777.05±252.54	0.563
Right thalamus volume, mm^2^	6696.93±830.19	6594.12±885.91	0.225
Right caudate volume, mm^2^	3373.49±565.75	3340.36±608.73	0.474
Right putamen volume, mm^2^	4743.54±678.03	4714.12±697.27	0.666
Right pallidum volume, mm^2^	1928.99±259.36	1922.71±272.97	0.812
Right cortex volume, mm^2^	216,046.23±23,917.16	212,025.16±26,239.96	0.104
Right cerebral white matter volume, mm^2^	222,783.05±29,373.45	217,819.36±30,845.32	0.096
Right mean cortical thickness, mm	2.37±0.12	2.37±0.11	0.800
Cortex volume, mm^2^	432,185.48±47,269.6	424,949.37±51,768.69	0.138
Subcortex gray volume, mm^2^	54,526.69±6033.69	54,063.54±6179.62	0.445
Total gray volume, mm^2^	585,324.16±59,164.42	576,922.21±63,115.96	0.164
Cerebral white matter volume, mm^2^	446,359.92±58,982.67	436,772.07±61,698.97	0.109
WM hyperintensities volume, mm^2^	3219.53±4544.25	3335.6±5123.64	0.510
Brain segmentation volume, mm^2^	1,089,199.77±112,197.33	1,067,993.91±118,965.54	0.110
eTIV, mm^2^	1,443,152.53±151,357.93	1,422,097.74±162,271.98	0.199
Brain segmentation volume to eTIV, %	75.67±5.19	75.37±6.11	0.590

*Abbreviations*: *CDR* Clinical Dementia Rating, *MMSE* Mini-Mental State Examination, *MoCA* Montreal Cognitive Assessment, *DST* Digit Span Test, *RAVLT* Rey Auditory Verbal Learning Test, *ROCF* Rey-Osterrieth Complex Figure Test, *TMT* Trail Making Test, *BNT* Boston Naming Test, *SDMT* Symbol Digit Modalities Test, *CDT* clock drawing test, *NPI* Neuropsychiatry Inventory, *WM* white matter, *eTIV* estimated total intracranial volume

Reading years and reading content had little impact on cognitive performance and brain structure (Supplemental Tables 1 and 2). Reading e-books showed no obvious cognitive benefits than paper books, and listening to audio-books performed better in MoCA, BNT, and SDMT (Supplemental Table 3).

**Fig. 3:**
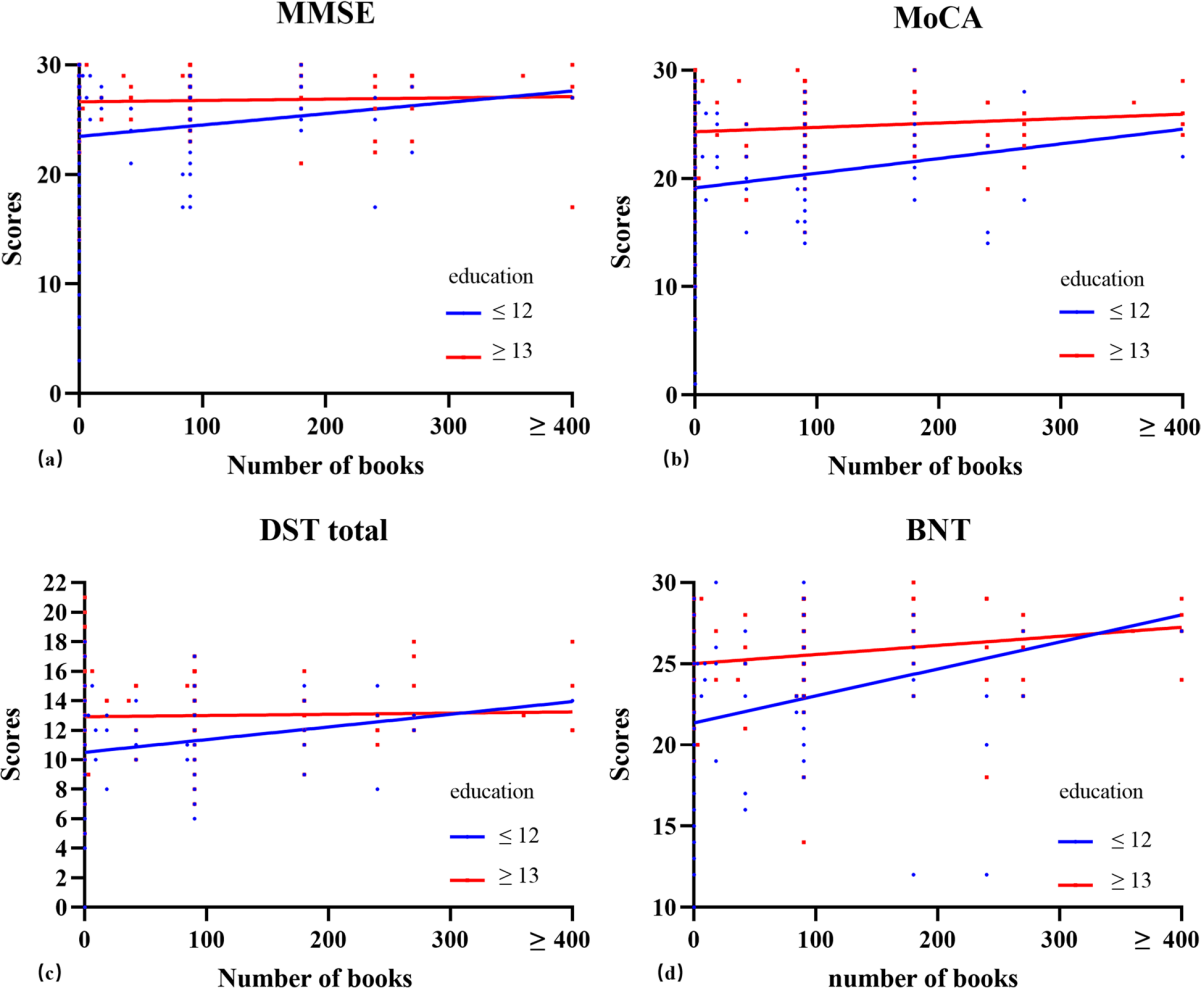
Cognitive performance associated with reading activities at different education levels. Shown is that by reading more books, participants with low education (blue line) could achieve similar or even better cognitive scores than those with high education (red line) in **a** MMSE, **b** MoCA, **c** DST, and **d** BNT. **a** MMSE (education ≤12: beta, 95% CI 0.1035 [0.0009, 0.0198], *P*=0.0316; education ≥13: beta, 95% CI 0.0012 [−0.0035, 0.0059], *P*=0.6113); **b** MoCA (education ≤12: beta, 95% CI 0.0136 [0.0031, 0.0241], *P*=0.0112; education ≥13: beta, 95% CI 0.0040 [−0.0020, 0.0100], *P*=0. 1856); **c** DST (education ≤12: beta, 95% CI 0.0086 [0.0035, 0.0136], *P*=0.0009; education ≥13: beta, 95% CI 0.0008 [−0.0037, 0.0053], *P*=0.7199); **d** BNT (education ≤12: beta, 95% CI 0.0166 [0.0076, 0.0256], *P*=0.0003; education ≥13: beta, 95% CI 0.0012 [0.0012, 0.0100], *P*=0.0371)

To assess if reading activities have a compensatory effect for low education, we divided participants into four groups: low education (educational years ≤12) with and without reading activities (groups 1 and 2), and high education (educational years ≥13) with and without reading activities (groups 3 and 4). Reading activities improved most cognitive tests (except RAVLT, ROCF copy, and TMT-B) in the low education group (group 1 better than group 2, Table 4). By reading more books, participants with low education could achieve similar or even better cognitive scores than those with high education in MMSE, MoCA, DST, and BNT (Fig. 3). In the high education groups, reading activities showed few effects probably due to ceiling effects (group 3 similar to group 4, Table 4).

**Table 4:** Cognitive performance and brain structure comparison by education level and reading activities

	Group 1 *n*=71	Group 2 *n*=223	*P*	Group 3 *n*=98	Group 4 *n*=67	*P*
Age	62.66±9.79	61.34±7.73	0.299	58.63±8.09	55.67±9.5	0.825
Gender female (*n*, %)	39, 54.9%	138, 61.9%	0.297	50, 51.0%	33, 49.3%	0.824
Education years	10.44±1.95	9.58±2.42	0.007	16.37±1.08	16.18±0.72	0.180
Global CDR score	0.14±0.23	0.24±0.44	0.323	0.08±0.20	0.13±0.28	0.236
MMSE	25.04±3.13	23.33±4.97	0.001	26.94±2.35	26.42±3.34	0.241
MoCA	21.26±4.05	18.9±5.37	0.001	24.9±2.82	24.22±4.52	0.280
DST total	11.59±2.27	10.45±2.53	0.001	12.82±2.26	13.21±3.19	0.387
RAVLT total learning	35.97±9.55	33.81±11.85	0.123	42.85±9.45	44.44±12.02	0.345
RAVLT long-delayed recall	6.07±3.43	6.03±3.79	0.931	8.73±3.29	8.53±3.88	0.726
ROCF copy	30.36±8.25	27.99±10.07	0.167	33.96±5.08	30.84±9.53	0.186
ROCF long-delayed recall	14.33±8.32	10.39±8.73	0.012	18.31±6.95	14.05±9.92	0.096
Stroop D time	18.19±7.84	23.31±17.69	0.001	15.91±4.35	16.75±8.88	0.419
Stroop W time	24.97±9.69	28.59±14.85	0.019	20.42±6	20.72±11.21	0.844
TMT-A time	50.89±25.93	62.56±33.49	0.003	39.39±16.34	43.62±40.19	0.419
TMT-B time	128.57±91.74	139.01±90.87	0.405	82.26±44.79	79.91±54.49	0.764
BNT	23.97±3.87	21.08±4.43	<0.001	25.77±2.81	24.96±2.45	0.057
SDMT	33.07±12.71	29.31±13.05	0.035	44.21±11.89	43.84±15.18	0.864
CDT	8.59±1.92	7.82±2.50	0.008	8.94±1.69	8.98±1.87	0.870
NPI	1.31±4.25	1.89±6.29	0.478	0.83±2.09	1.65±4.97	0.206
Brain structure						
Left hippocampal volume, mm^2^	3391.25±442.13	3372.73±419.86	0.759	3580.93±455.11	3587.09±411.2	0.93
Left amygdala volume, mm^2^	1487.93±400.96	1494.7±333.99	0.889	1444.95±319.72	1443.14±289.65	0.971
Left thalamus volume, mm^2^	6927.86±912.39	6868.82±894.71	0.635	6844.53±902.39	6902.71±1077.97	0.713
Left caudate volume, mm^2^	3274.19±590.97	3326.22±577.32	0.518	3347.72±546.78	3204.22±589.96	0.118
Left putamen volume, mm^2^	4613.36±742.16	4634.88±700.9	0.827	4647.23±600.29	4543.36±777.51	0.344
Left pallidum volume, mm^2^	1958.16±291.24	1922.58±236.29	0.307	1926.63±253.55	1896.81±246.62	0.463
Left cortex volume, mm^2^	215,000.93±24,471.4	216,524.19±23,482.27	0.643	212,619.25±24,800.12	213,376.89±27,526.83	0.857
Left cerebral white matter volume, mm^2^	225,861.59±35,115.03	222,804.26±27,795.56	0.51	217,545.76±29,530.28	221,041.16±33,286.15	0.488
Left mean cortical thickness, mm	2.37±0.12	2.36±0.11	0.34	2.38±0.11	2.38±0.1	0.796
Right hippocampal volume, mm^2^	3533.42±467.04	3449.84±500.66	0.205	3695.9±417.79	3720.84±445.47	0.723
Right amygdala volume, mm^2^	1638.97±387.11	1671.78±333.05	0.495	1646.86±334.66	1639.52±320.52	0.89
Right thalamus volume, mm^2^	6739.93±910.74	6682.39±802.96	0.617	6571.9±886.17	6627.11±891.5	0.701
Right caudate volume, mm^2^	3326.03±602.96	3389.53±553.2	0.418	3396.05±628.68	3257.71±572.69	0.161
Right putamen volume, mm^2^	4702.98±639.94	4757.25±691.4	0.564	4723.38±655.7	4700.38±759.94	0.839
Right pallidum volume, mm^2^	1939.62±285.7	1925.39±250.47	0.692	1935.71±280.49	1903.42±262.42	0.466
Right cortex volume, mm^2^	214,709.43±24,120.85	216,498.29±23,889.7	0.589	211,079.33±26,149.49	213,429.13±26,517.63	0.581
Right cerebral white matter volume, mm^2^	225,512.97±34,160.89	221,859.89±27,599.24	0.42	215,805.29±29,593.22	220,808.98±32,622.88	0.317
Right mean cortical thickness, mm	2.37±0.12	2.35±0.11	0.142	2.38±0.11	2.37±0.11	0.427
Cortex volume, mm^2^	429,710.36±48,310.18	433,022.48±47,001.73	0.613	423,698.58±50,582.34	426,806.01±53,831.66	0.712
Subcortex gray volume, mm^2^	54,504.63±6480.09	54,534.15±5891.59	0.972	54,151.2±5827.67	53,933.42±6713.47	0.828
Total gray volume, mm^2^	582,730.42±62,282.68	586,201.27±58,202.35	0.672	576,181.75±62,013.7	578,021.34±65,196.6	0.858
Cerebral white matter volume, mm^2^	451,374.56±69,166.21	444,664.14±55,202.29	0.463	433,351.05±58,867.31	441,850.14±65,823.54	0.396
WM hyperintensities volume, mm^2^	3602.52±4963.72	3090.02±4398.62	0.416	3925.16±6044.47	2460.48±3165.75	0.048
Brain segmentation volume, mm^2^	1,093,169.74±123,689.54	1,087,857.27±108,324.4	0.733	1,065,497.73±113,587.95	1,071,699.19±127,357.94	0.748
eTIV, mm^2^	1,440,816.13±159,459	1,443,942.62±148,911.25	0.882	1,422,070.61±165,054.25	1,422,138.01±159,346.57	0.998
Brain segmentation volume to eTIV, %	76.03±5.26	75.55±5.18	0.507	75.26±6.19	75.54±6.03	0.783

Four groups: low education (educational years ≤12) with and without reading activities (groups 1 and 2) and high education (educational years ≥13) with and without reading activities (groups 3 and 4)

*Abbreviations*: *CDR* Clinical Dementia Rating, *MMSE* Mini-Mental State Examination, *MoCA* Montreal Cognitive Assessment, *DST* Digit Span Test, *RAVLT* Rey Auditory Verbal Learning Test, *ROCF* Rey-Osterrieth Complex Figure Test, *TMT* Trail Making Test, *BNT* Boston Naming Test, *SDMT* Symbol Digit Modalities Test, *CDT* clock drawing test, *NPI* Neuropsychiatry Inventory, *WM* white matter, *eTIV* estimated total intracranial volume

The linear regression related to hippocampal volume on either side showed that years of education influenced hippocampal volume with beta1 14.999 [4.906, 25.092], *P*=0.004 and beta2 15.816 [4.949, 26.683], *P*=0.004 (Table 5), regardless of age and sex.

**Table 5:** The linear regression of education and hippocampal volume

	Model 1, beta, 95% CI	*P*	Model 2, beta, 95% CI	*P*
Years of education	14.999, [4.906, 25.092]	0.004	15.816, [4.949, 26.683]	0.004
Education ≥13	23.020, [3.868, 42,172]	0.019	22.114, [1.476, 42.753]	0.036

Model 1, data of left hippocampal volume were analyzed as results, age, and sex were in linear regression

Model 2, data of right hippocampal volume were analyzed as results, age, and sex were in linear regression

*Abbreviations*: *CI* confidence interval

## Discussion

In this community-based subjectively cognitively normal population, participants with regular reading activities showed better cognitive performance in overall cognitive abilities, attention, memory, language, visuospatial and executive function. This effect is independent of brain volume, especially hippocampal volume. A prospective cohort study showed that increased participation in cognitive activities (including reading) was associated with better memory [21]. Although reading activities involve multiple brain areas, subgroup analysis of the FINGER study has shown that the multi-domain intervention has no effects on brain volume, cortical thickness, and white matter lesion [22].

Education was related to cognition across all tested domains. Reading is associated with all tested domains controlled with age and sex. However, when education was included in the analysis, the effect of reading on cognitive assessment weakened, indicating a stronger correlation between education level and cognition. Reading is a complex task that involves various brain areas, including the insular and frontal opercular cortex, lateral temporal cortex, and early auditory cortex with the positive reaction and inferior temporal and motor cortex with the negative reaction [23]. However, we did not see a difference in the cortical thickness and the hippocampus between reading and non-reading groups. This suggests that reading activities may help to improve cognitive function in participants with low education level (≤12 years) independent of brain volume. In some cognitive domains, the cognitive performance gap caused by education level is decreased with the increase in reading activities. Reading is a good way to fill the cognitive gap brought about by lack of education, especially in language, non-verbal memory, and executive function.

In this study, participants with high education level had higher hippocampal volume. Larger hippocampal volumes may be associated with higher intelligence quotient (IQ), practice in hippocampus-related function (e.g., learning and memory), lifestyle, and medial/historical factors (neurotoxic effects of obesity, diabetes mellitus, hypertension, hypoxic brain injury, obstructive sleep apnea, bipolar disorder, clinical depression, and head trauma) [24]. Higher education level is favorable to the neurological task performance [10, 12, 25–27], but not to AD-related pathology [28].

MMSE and MoCA are screening tests for cognition. Their cut-off scores are based on education levels. In this study, participants with low education but reading more books showed no difference in MMSE and MoCA compared with participants with high education level. It suggests that people with low education but who read a lot probably should be screened at the same level as those who are more educated.

Audio devices are a new form of reading activities and have become popular. It is suggested that audiobooks are probably better than non-audio books at improving cognitive function. Young children learned more words from the e-book and from the audio narrator than print books [29]. Different types of books may influence the ability to retrieve information. Listening to audiobooks may stimulate more brain areas to have positive effects on cognition, especially memory and executive function. Since poor vision is not uncommon in the elderly, audiobooks are a better tool for old people to enjoy reading activities.

The strength of this study is a large community-based cohort with detailed neuropsychological testing batteries and brain MRI analysis. But the study has several limitations. First, this is an observational, cross-sectional study. *Correlation does not imply causation*. To study the causative effect of reading activities on cognitive function, a randomized clinical trial is warranted. Participants with certain education levels would be assigned with different reading activities. Other intellectual activities besides leisure reading would also be taken into account. Second, we enrolled participants with subjectively normal cognitive function to represent community-based cohorts. The average CDR was 0.18 although a few participants with a CDR more than 0.5. Ongoing longitudinal follow-ups will allow us to assess the relationship between risk/protective factors and the conversion to dementia. Third, all participants were enrolled from northern China. There is likely a difference in culture, education, and environmental factors among different regions in China. To expand population sampling is needed in future studies. Fourth, higher education level is associated with larger hippocampal volumes. One explanation is that education may stimulate the growth and development of the hippocampus. Alternatively, people with larger hippocampal volume may have a better chance to acquire higher education. Fifth, the study may have a recall bias since reading activities were recorded by self-reported questionnaires. People might under- or overestimate the books they read. Objective measures (e.g., a shopping receipt of purchased books) may help to validate the finding. Finally, reading activities as measured by reading books are mainly leisure reading. It does not take into account of all activities related to intellectual activities. Individuals who do a lot of reading or research at work but have little time in reading books outside of work may be underestimated in reading activities.

## Conclusions

Participants in reading groups with less education (educational years ≤12) had better cognitive performance than the ones in non-reading groups. Education affects more than reading habits in every cognitive domain and in hippocampal volume.

## Supplementary Information

**Additional file 1: Supplemental Table 1.** Cognitive performance of participants having different reading years.

**Additional file 2: Supplemental Table 2.** Cognitive performance of participants reading different content.

**Additional file 3: Supplemental Table 3.** Cognitive performance of participants using different reading materials.

## Data Availability

SL and JS had full access to all of the data in the study and takes responsibility for the integrity of the data and the accuracy of the data analysis.

## Abbreviations

CANDOR: China National Clinical Research Center Alzheimer's Disease and Neurodegenerative Disorder Research
FINGER: Finnish Geriatric Intervention Study to Prevent Cognitive Impairment and Disability
AD: Alzheimer’s disease
MRI: Magnetic resonance imaging
NC: Normal cognition
MCI: Mild cognitive impairment
MMSE: Mini-Mental State Examination
CDR: Clinical Dementia Rating
MoCA: Montreal Cognitive Assessment
RAVLT: Rey Auditory Verbal Learning Test
ROCF: Rey-Osterrieth Complex Figure Test
TMT: Trail Making Test
CDT: Clock drawing test
BNT: Boston Naming Test
DST: Digit Span Test
SDMT: Symbol Digit Modalities Test
NPI: Neuropsychiatry Inventory
SD: Standard deviations
OR: Odds ratio
CI: Confidence interval
WM: White matter
eTIV: Estimated total intracranial volume
