# Supplementary material for: Reading activities compensate for low education-related cognitive deficits
Source: Alzheimers Res Ther. 2022 Oct 14;14:156. doi: 10.1186/s13195-022-01098-1 (PMC9563722; doi:10.1186/s13195-022-01098-1)
Supplement: Supplementary file 1 — Additional file 1: Supplemental Table 1. Cognitive performance of participants having different reading years. [file 13195_2022_1098_MOESM1_ESM.docx]

**Supplemental Table 1：Cognitive performance of participants having different reading years**

|  | Years of reading <5 years  n=27 | Years of reading ≥5 years  n=142 | P |
| --- | --- | --- | --- |
| Average age | 58.59±9.71 | 60.65±8.9 | 0.278 |
| Gender female (n, %) | 18, 66.7% | 71, 50.0% | 0.112 |
| Years of education | 12.37±3.53 | 14.16±3.19 | 0.009 |
| Global CDR score | 0.11±0.21 | 0.11±0.21 | 0.903 |
| MMSE | 25.88±2.98 | 26.2±2.83 | 0.609 |
| MoCA | 22.77±4.02 | 23.49±3.79 | 0.377 |
| DST total | 12.31±2.11 | 12.3±2.38 | 0.992 |
| RAVLT total learning | 39.27±11.33 | 40.11±9.84 | 0.695 |
| RAVLT long delayed recall | 6.5±3.78 | 7.83±3.53 | 0.082 |
| ROCF copy | 32.07±6.37 | 32.38±7.03 | 0.871 |
| ROCF long delay recall | 14.57±10.12 | 16..82±7.38 | 0.304 |
| Stroop D time | 16.54±5.93 | 16.92±6.19 | 0.774 |
| Stroop W time | 23.73±10.76 | 22.06±7.46 | 0.331 |
| TMT-A time | 44.85±28.11 | 44.06±20.27 | 0.864 |
| TMT-B time | 95.27±68.64 | 102.7±72.64 | 0.629 |
| BNT | 23.92±3.35 | 25.22±3.38 | 0.074 |
| SDMT | 39.42±13.59 | 39.6±13.4 | 0.951 |
| CDT | 8.62±1.9 | 8.82±1.78 | 0.587 |
| NPI | 1.33±2.18 | 0.97±3.33 | 0.589 |

Abbreviations: CDR, Clinical Dementia Rating. MMSE, Minimum Mental State Examination. MoCA, Montreal Cognitive Assessment. DST, Digit Span Test, RAVLT, Rey Auditory Verbal Learning Test. ROCF, Rey-Osterrieth Complex Figure Test. TMT, Trail Making Test. BNT, Boston Naming Test. SDMT, Symbol Digit Modalities Test. CDT, Clock drawing test. NPI, Neuropsychiatry Inventory.
