# Supplementary material for: Reading activities compensate for low education-related cognitive deficits
Source: Alzheimers Res Ther. 2022 Oct 14;14:156. doi: 10.1186/s13195-022-01098-1 (PMC9563722; doi:10.1186/s13195-022-01098-1)
Supplement: Supplementary file 2 — Additional file 2: Supplemental Table 2. Cognitive performance of participants reading different content. [file 13195_2022_1098_MOESM2_ESM.docx]

**Supplemental Table 2：Cognitive performance of participants reading different content**

|  | Literature  n=14 | Non literature  n=124 | Both type  n=12 | P | Reading web novel  n=30 | Reading other books  n=139 | P |
| --- | --- | --- | --- | --- | --- | --- | --- |
| Average age | 64.09±7.29 | 60.27±8.89 | 58.94±8.07 | 0.238 | 59.32±10.77 | 12.39±3.59 | 0.486 |
| Gender female (n, %) | 9, 64.3% | 63, 50.8% | 8, 66.7% | 0.397 | 18, 60% | 70, 50.4% | 0.178 |
| Years of education | 15.64±1.21 | 13.76±3.43 | 15±2.57 | 0.045 | 12.39±3.59 | 14.14±3.18 | 0.010 |
| Global CDR score | 0.04±0.13 | 0.12±0.21 | 0 | 0.070 | 0.16±0.27 | 0.1±0.2 | 0.251 |
| MMSE | 26.73±2.41 | 25.95±2.79 | 26.77±3.01 | 0.290 | 26.32±3.48 | 26.07±2.71 | 0.665 |
| MoCA | 23.73±2.87 | 22.82±4.13 | 25.06±2.61 | 0.016 | 23.54±4.19 | 23.3±3.76 | 0.771 |
| DST total | 13±1.84 | 12.14±2.48 | 12.68±2.31 | 0.343 | 12.86±1.76 | 12.17±2.43 | 0.156 |
| RAVLT total learning | 40.82±8.05 | 39.93±10.35 | 39.94±8.91 | 0.960 | 42.43±9.32 | 39.4±10.2 | 0.148 |
| RAVLT long delayed recall | 7.36±2.77 | 7.55±3.78 | 8±3.24 | 0.805 | 8.36±3.53 | 7.41±3.56 | 0.198 |
| ROCF copy | 35.3±1.06 | 18.2±5.34 | 18±5.98 | 0.116 | 33.15±2.73 | 32.09±7.52 | 0.569 |
| ROCF long delay recall | 18±5.98 | 19.1±2.13 | 3462.63±438.2 | 0.707 | 15.65±8.63 | 16.61±7.78 | 0.650 |
| Stroop D time | 0.73±1.85 | 0.17±1.01 | 0±0 | 0.115 | 16.18±5.42 | 17.03±6.31 | 0.507 |
| Stroop W time | 0.64±1.8 | 0.21±0.91 | 0.03±0.18 | 0.170 | 22.36±8.82 | 22.38±7.95 | 0.990 |
| TMT-A time | 46.45±26.52 | 44.84±20.26 | 41.32±19.37 | 0.657 | 46.64±28.31 | 43.85±20.13 | 0.535 |
| TMT-B time | 109.27±84.22 | 106.13±75.76 | 92.23±57.47 | 0.624 | 111.04±104.06 | 100.25±64.18 | 0.472 |
| BNT | 25.45±3.53 | 24.6±3.62 | 26.1±2.72 | 0.096 | 25.18±3.28 | 24.96±3.44 | 0.762 |
| SDMT | 42.09±11.55 | 39.36±13.88 | 41.06±10.28 | 0.693 | 40.14±14.17 | 39.35±13.34 | 0.776 |
| CDT | 9.55±0.82 | 8.56±1.97 | 8.9±1.56 | 0.192 | 9.39±1.59 | 8.67±1.82 | 0.051 |
| NPI | 0.91±1.58 | 0.97±3.52 | 0.77±2.32 | 0.955 | 2.89±6.65 | 0.67±1.64 | 0.089 |

Abbreviations: CDR, Clinical Dementia Rating. MMSE, Minimum Mental State Examination. MoCA, Montreal Cognitive Assessment. DST, Digit Span Test, RAVLT, Rey Auditory Verbal Learning Test. ROCF, Rey-Osterrieth Complex Figure Test. TMT, Trail Making Test. BNT, Boston Naming Test. SDMT, Symbol Digit Modalities Test. CDT, Clock drawing test. NPI, Neuropsychiatry Inventory.
