# Supplementary material for: Reading activities compensate for low education-related cognitive deficits
Source: Alzheimers Res Ther. 2022 Oct 14;14:156. doi: 10.1186/s13195-022-01098-1 (PMC9563722; doi:10.1186/s13195-022-01098-1)
Supplement: Supplementary file 3 — Additional file 3: Supplemental Table 3. Cognitive performance of participants using different reading materials. [file 13195_2022_1098_MOESM3_ESM.docx]

**Supplemental Table 3：Cognitive performance of participants using different reading materials**

|  | Reading paper books  n=64 | Reading E-books  n=66 | Reading both types  n=39 | P | Audio books  n=51 | Non-audio books  n=118 | P |
| --- | --- | --- | --- | --- | --- | --- | --- |
| Average age | 59.33±8.49 | 61.86±9.24 | 59.5±9.26 | 0.236 | 59.76±9.61 | 60.57±8.81 | 0.597 |
| Gender female, (n, %) | 32, 50% | 39, 59.1% | 19, 48.7% | 0.378 | 28, 54.9% | 61, 51.7% | 0.702 |
| Years of education | 13.86±3.22 | 13.92±3.51 | 14.14±3.16 | 0.919 | 14.29±3.19 | 13.69±3.34 | 0.280 |
| Global CDR score | 0.14±0.22 | 0.09±0.21 | 0.10±0.21 | 0.371 | 0.09±0.19 | 0.11±0.22 | 0.464 |
| MMSE | 26.24±2.88 | 25.57±3 | 26.92±2.43 | 0.073 | 26.69±2.79 | 25.91±2.86 | 0.107 |
| MoCA | 23.24±3.97 | 22.79±3.84 | 24.53±3.53 | 0.094 | 24.57±3.5 | 22.86±3.86 | 0.007 |
| DST total | 12.06±2.36 | 12.13±2.38 | 13±2.24 | 0.126 | 12.67±2.32 | 12.15±2.34 | 0.184 |
| RAVLT learn total | 40.84±9.13 | 38.95±11.07 | 40.94±9.42 | 0.490 | 41.2±9.94 | 39.45±10.11 | 0.303 |
| RAVLT long delayed recall | 8.32±3.06 | 6.78±3.76 | 8±3.77 | 0.040 | 8.2±3.79 | 7.38±3.49 | 0.174 |
| ROCF copy | 33.4±3.49 | 31.55±7.69 | 31.81±9.48 | 0.486 | 32.52±8.3 | 32.26±6.25 | 0.861 |
| ROCF delay recall | 18.17±5.59 | 15.1±9.18 | 16.79±7.8 | 0.227 | 17.77±7.4 | 15.92±7.99 | 0.271 |
| Stroop D time | 17.83±7.14 | 16.87±5.38 | 16.28±4.29 | 0.425 | 16.61±4.74 | 16.97±6.67 | 0.728 |
| Stroop W time | 23.02±8.25 | 23.65±8.22 | 20.31±5.41 | 0.107 | 21.06±7.44 | 22.86±8.27 | 0.182 |
| TMT-A time | 43.17±20.52 | 47.44±23.8 | 42.69±19.21 | 0.442 | 42.43±22.76 | 44.94±21.08 | 0.490 |
| TMT-B time | 96.94±54.41 | 116.24±78.3 | 92.25±86.65 | 0.189 | 90.27±79.81 | 106.47±67.93 | 0.180 |
| BNT | 24.97±3.32 | 24.3±3.42 | 26.06±3.38 | 0.048 | 25.94±3.13 | 24.62±3.45 | 0.020 |
| SDMT | 39.33±12.42 | 37.98±15.02 | 43.33±10.68 | 0.148 | 43.1±12.67 | 38.03±13.46 | 0.024 |
| CDT | 8.86±2.05 | 8.54±1.58 | 9.11±1.67 | 0.296 | 9.06±1.62 | 8.68±1.86 | 0.203 |
| NPI | 0.9±2.22 | 1.11±2.22 | 1.25±5.52 | 0.868 | 1.52±5.12 | 0.82±1.81 | 0.351 |

Abbreviations: CDR, Clinical Dementia Rating. MMSE, Minimum Mental State Examination. MoCA, Montreal Cognitive Assessment. DST, Digit Span Test, RAVLT, Rey Auditory Verbal Learning Test. ROCF, Rey-Osterrieth Complex Figure Test. TMT, Trail Making Test. BNT, Boston Naming Test. SDMT, Symbol Digit Modalities Test. CDT, Clock drawing test. NPI, Neuropsychiatry Inventory.
